# Oxygen saturation ranges for healthy newborns within 24 hours at 1800 m

**DOI:** 10.1136/archdischild-2016-311813

**Published:** 2017-02-02

**Authors:** Melissa C Morgan, Beth Maina, Mary Waiyego, Catherine Mutinda, Jalemba Aluvaala, Michuki Maina, Mike English

**Affiliations:** 1 Department of Paediatrics, University of California San Francisco, San Francisco, California, USA; 2 Pumwani Maternity Hospital, Nairobi, Kenya; 3 Department of Paediatrics and Child Health, University of Nairobi, Nairobi, Kenya; 4 KEMRI-Wellcome Trust Research Programme, Nairobi, Kenya; 5 Nuffield Department of Medicine & Paediatrics, University of Oxford, Oxford, UK

**Keywords:** pulse oximetry, oxygen saturation, SpO2, infant, preterm

## Abstract

There are minimal data to define normal oxygen saturation (SpO_2_) levels for infants within the first 24 hours of life and even fewer data generalisable to the 7% of the global population that resides at an altitude of >1500 m. The aim of this study was to establish the reference range for SpO_2_ in healthy term and preterm neonates within 24 hours in Nairobi, Kenya, located at 1800 m. A random sample of clinically well infants had SpO_2_ measured once in the first 24 hours. A total of 555 infants were enrolled. The 5th–95th percentile range for preductal and postductal SpO_2_ was 89%–97% for the term and normal birthweight groups, and 90%–98% for the preterm and low birthweight (LBW) groups. This may suggest that 89% and 97% are reasonable SpO_2_ bounds for well term, preterm and LBW infants within 24 hours at an altitude of 1800 m.

**What is already known on this topic?**

- Proper use of pulse oximetry to guide oxygen therapy relies on knowledge of normal oxygen saturation (SpO_2_) values for a given population.
- Based on available evidence, it appears that well term infants born at moderate altitude should have SpO_2_ values of ≥92% *after* the first 24 hours.
- The optimal SpO_2_ for well newborns within 24 hours of life and for well preterm infants born at moderate altitude is unknown.

**What this study adds?**

- This is the largest study of SpO_2_ ranges for well preterm infants at moderate altitude.
- This study suggests that a SpO_2_ range of 89%–97% is suitable for well term and preterm infants within 24 hours at 1800 m.
- This study provides important data about SpO_2_ in the first 24 hours, which are useful as discharge postdelivery occurs within 24 hours in many low-income countries.

## Introduction

There is a large burden of neonatal mortality in low-income countries (LIC). Hypoxaemia occurs in a substantial portion of hospitalised neonates, and is significantly associated with mortality. Pulse oximetry is a non-invasive method of measuring the oxygen saturation of haemoglobin, and has become a critical tool in determining need for oxygen in sick newborns. Routine use may also aid identification of infants with clinically unrecognised respiratory abnormalities in LIC, where discharge often occurs within the first 24 hours of life.

Use of pulse oximetry relies on knowledge of normal oxygen saturation (SpO_2_) values. It is important to define normal SpO_2_ levels for neonates of different gestational ages (GA) at different time points after birth. SpO_2_ values prior to 24 hours are lower and more variable than those seen after 24 hours. Similarly, SpO_2_ values may be lower at higher altitudes, such as in Kenya where 20% of the population resides above 1500 m. Thilo *et al*^1^ found that mean SpO_2_ was 92%–93% at 24–48 hours among well term neonates at 1610 m. Ravert *et al*^2^ found mean SpO_2_ of 95%–97% at ∼1371 m and 94%–95% at ∼2073 m among well term newborns during the first 72 hours. Three studies evaluated SpO_2_ in healthy preterm infants. Ng *et al*^3^ (N=33) found mean SpO_2_ of 97% (median GA 33 weeks, median age 14 days); Harigopal *et al*^4^ (N=43) found median SpO_2_ of 95% (median GA 33 weeks, median age 14 days) and Richard *et al*^5^ (N=55) found median SpO_2_ of 99% (mean GA 35, mean age 1 day). All of these studies were conducted at sea level.^3–5^ Specific gaps remain with sparse data for newborns within the first 24 hours or for well preterm infants, with no published reference ranges for preterm infants born at increased altitude. The aim of this study was to establish within 24 hours the reference interval for preductal and postductal SpO_2_ in healthy term and preterm neonates at 1800 m.

## Methods

### Participants and setting

This study was conducted at Pumwani Hospital in Nairobi, Kenya, located at 1800 m. The hospital provides care to ∼22 000 women and their infants each year. A 120-bed nursery provides care for all infants requiring medical attention. Inclusion criteria included being born alive at Pumwani Hospital during the study period and appearing well as defined by (1) normal vital signs (heart rate 110–180 beats/min, respiratory rate 30–60 breaths/min, temperature 36.5°C–37.5°C), (2) absence of respiratory distress and (3) absence of other signs of illness (eg, poor suck, lethargy). Infants were excluded if they required admission for any reason other than observation or if they were transferred to another facility within 4 hours for anomalies or severe medical problems.

### Sampling approach

We aimed to enrol 800 well neonates, stratified to include 400 term (≥37 weeks) and 400 preterm (<37 weeks) neonates. GA was based on last menstrual period (LMP), and Ballard examination was conducted when LMP was unknown or incongruent with appearance. We screened all preterm neonates and a random proportion of term neonates.

### Procedures

Enrolled infants underwent testing once between 1 and 24 hours with the Lifebox oximeter, which was developed for low-resource settings by WHO and World Federation of Societies for Anaesthesiologists (Acare Technology, New Taipei City, Taiwan). The measurement was recorded when there was a good waveform for ≥15 s and SpO_2_ was stable over that period.

### Analysis

We determined mean, SD, median, IQR and 5th–95th percentile range of preductal and postductal SpO_2_ measurements, stratifying by GA (term or preterm), birth weight (normal (NBW), ≥2.5 kg or low (LBW), <2.5 kg) and postnatal age (0–6, 6–12, 12–18, 18–24 hours). Statistical analyses were conducted using Stata V.13 (StataCorp, College Station, Texas, USA).

### Ethical aspects

Written informed consent was obtained from parents/guardians. Ethical approval was received from the University of California, San Francisco and the Kenya Medical Research Institute-Wellcome Trust Research Programme.

## Results

A total of 555 infants were enrolled between January and December 2015. The mean GA was 38 weeks (SD 2.4), mean birth weight was 2.94 kg (SD 0.53), 50% were male and 93% were delivered vaginally. Among preterm infants, median GA was 35 weeks (range 27–36).

Mean preductal SpO_2_ was 93%–94% across all GA and birthweight groups. Mean postductal SpO_2_ was 93%–94% for all GA groups, 93% for birth weights ≥2.5 kg and 95% for <2.5 kg. Median preductal and postductal SpO_2_ values were similar (table 1). The 5th–95th percentile range for preductal and postductal SpO_2_ was between 89% and 97% for the term and NBW groups, and between 90% and 98% for the preterm and LBW groups.

**Table 1:** Preductal and postductal SpO_2_ by gestational age and birth weight

	Gestational age		Birth weight	
	Term (n=420)	Preterm (n=135)	≥2.5 kg (n=456)	<2.5 kg (n=99)
Preductal SpO_2_, %				
Mean, SD	93 (2.8)	94 (2.8)	93 (2.8)	94 (2.6)
Median, IQR	94 (91–95)	94 (92–96)	94 (91–95)	94 (92–96)
5%–95% range	89–97	90–98	89–97	90–98
Postductal SpO_2_, %				
Mean, SD	93 (2.5)	94 (2.5)	93 (2.6)	95 (2.3)
Median, IQR	94 (92–95)	95 (93–96)	94 (92–95)	95 (93–96)
5%–95% range	89–97	90–98	89–97	90–98

SpO_2_, normal oxygen saturation.

When stratified by postnatal age, results were similar (table 2).

**Table 2:** Preductal and postductal SpO_2_ by postnatal age

	Postnatal age (hours of life)			
	0–6 hours (n=109)	6–12 hours (n=145)	12–18 hours (n=147)	18–24 hours (n=137)
Preductal SpO_2_, %				
Mean, SD	94 (2.9)	93 (2.9)	93 (2.5)	93 (2.9)
Median, IQR	95 (92–96)	94 (91–95)	93 (91–95)	93 (91–95)
5%–95% range	90–98	89–98	89–97	88–98
Postductal SpO_2_, %				
Mean, SD	94 (2.7)	93 (2.4)	94 (2.4)	93 (2.5)
Median, IQR	95 (92–96)	94 (92–95)	94 (92–95)	93 (92–95)
5%–95% range	90–98	90–97	90–97	88–97

SpO_2_, normal oxygen saturation.

**Figure 1:**
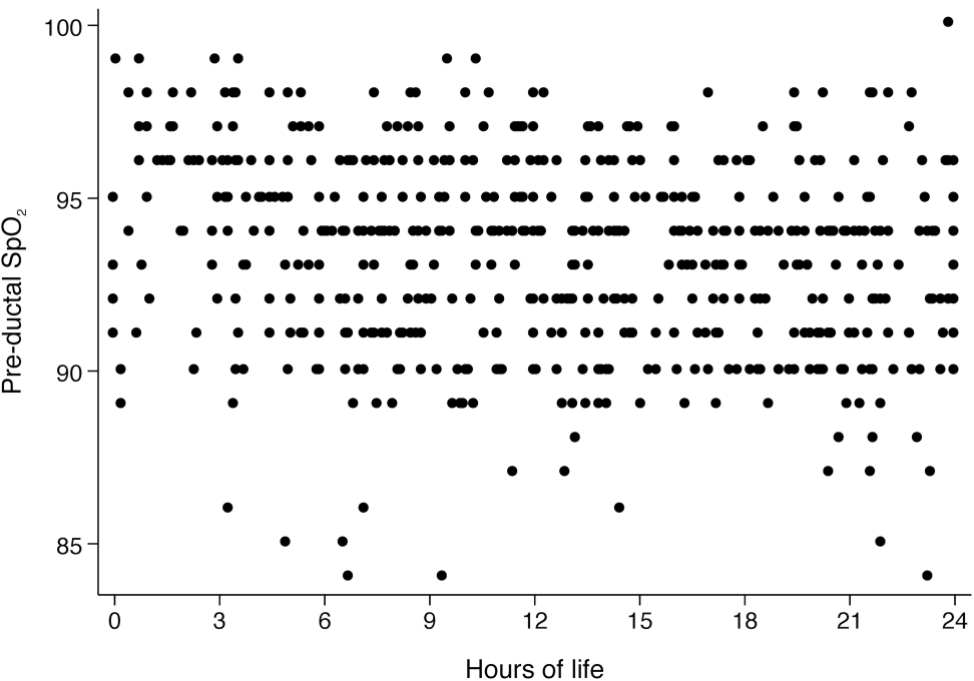
Preductal normal oxygen saturation (SpO2) by hours of life.

**Figure 2:**
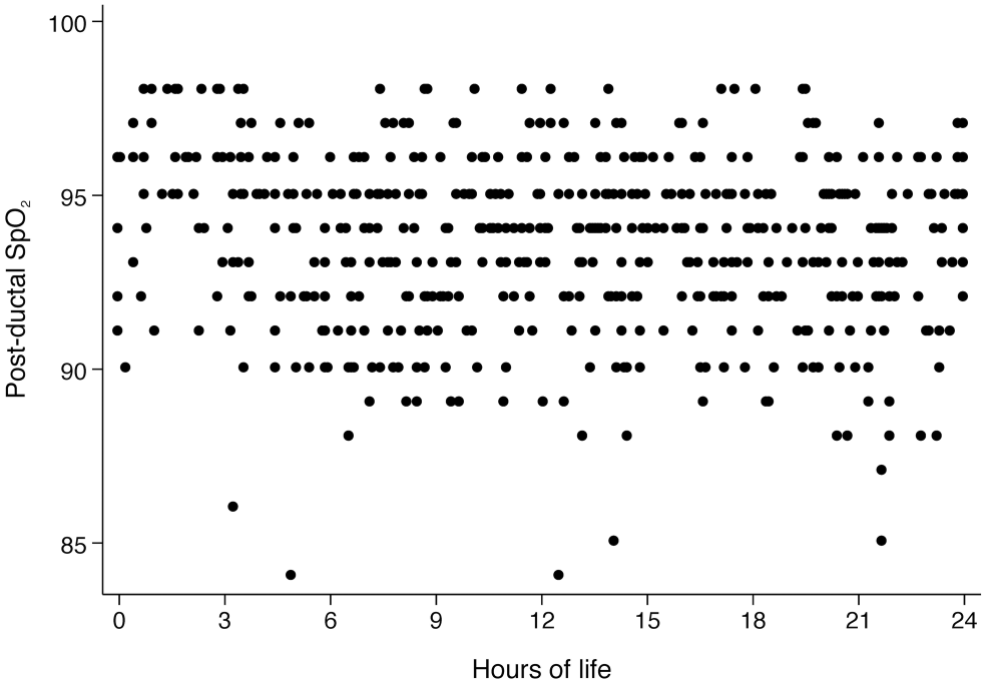
Postductal normal oxygen saturation (SpO2) by hours of life.

Scatter plots of preductal and postductal SpO_2_ by hours are shown in figures 1 and 2, respectively, and also suggest absence of change across time.

## Discussion and conclusion

Data are sparse to inform our understanding of oxygen saturation norms for newborns within the first 24 hours and for well preterm infants. We found that mean preductal and postductal SpO_2_ was 93%–95% in all groups within 24 hours. This is similar to findings among well term infants in studies conducted by Thilo *et al*^1^ and Ravert *et al*.^2^ The mean and median SpO_2_ for preterm infants were only slightly lower than those found by Ng *et al*,^3^ Harigopal *et al*^4^ and Richard^5^ at sea level despite the early postnatal age and increased altitude in our study. We found that the 5th–95th percentile ranges for preductal and postductal SpO_2_ were 89%–97% for the term and NBW groups and 90%–98% for the preterm and LBW groups. Thilo *et al* reported a 95% CI of 89% to 97%,^1^ which corresponds with our findings.

This study has limitations. Our findings for preterm infants are based on a small sample of 135 infants. It was difficult to recruit well preterm infants as many such infants were admitted for medical therapy, making them ineligible. Additional research about SpO_2_ reference ranges in preterm infants born at a variety of altitudes is needed. If methods and timing for collecting measurements were sufficiently similar, these data could make a considerable contribution to a pooled sample for meta-analysis.

In this study, GA was based on LMP with Ballard examination conducted when LMP was unknown or incongruent with appearance. LMP is subject to bias and Ballard may differ from ultrasound by 1 to 2 weeks. However, ultrasound is unavailable in many LIC facilities due to cost and need for skilled sonographers. The Lifebox oximeter is not motion-resistant and has not been validated in neonates. To ensure we obtained the most accurate measurement possible, we recorded measurements only when there was a good waveform for ≥15 s and SpO_2_ was stable over that period.

Reports suggest that pulse oximetry use can decrease mortality in children with unrecognised hypoxaemia. In newborns, it is increasingly being used for predischarge screening to diagnose occult respiratory and cardiac disease. This study suggests that 89% and 97% may be reasonable SpO_2_ bounds for well term, preterm and LBW infants within 24 hours at 1800 m, although we lack outcome data that would confirm infants in this study remained well. This study provides important data about SpO_2_ within the first 24 hours, which are useful as discharge postdelivery is rapid and typically without skilled assessment by a clinician or nurse in many LIC settings.
